# In Vivo Confocal Microscopy in Avellino Corneal Dystrophy

**DOI:** 10.7759/cureus.67311

**Published:** 2024-08-20

**Authors:** Özge Oztürk, Sina Hakami, Artémise Dugauquier, Pauline Le Roux

**Affiliations:** 1 Department of Ophthalmology, Erasmus Hospital, Brussels, BEL

**Keywords:** corneal dystrophy type 2, epithelial-stromal corneal dystrophy, granular-lattice corneal dystrophy, avellino corneal dystrophy, corneal dystrophy, in vivo confocal microscopy

## Abstract

Corneal dystrophies are a group of rare genetic eye disorders characterized by the accumulation of abnormal material in different layers of the cornea, potentially leading to vision impairment.

In vivo confocal microscopy (IVCM) is an emerging non-invasive imaging and diagnostic tool that helps study the ocular surface microstructure.

This case report examines the clinical characteristics of Avellino corneal dystrophy in a young patient through the use of slit lamp examination, IVCM, and optical coherence tomography (OCT) in order to assess the effectiveness of these non-invasive tests as diagnostic tools.

## Introduction

Corneal dystrophies are defined as a group of rare genetic eye disorders characterized by the accumulation of abnormal material in different layers of the cornea. Depending on the affected layer, they are classified as epithelial-subepithelial dystrophies, epithelial-stromal dystrophies, stromal dystrophies, and endothelial dystrophies [1].

Avellino corneal dystrophy, also known as granular corneal dystrophy type II (GCD2), is an autosomal dominant inherited disorder that belongs to the epithelial-stromal dystrophies. The pathophysiology of the disease is explained by the mutation of the transforming growth factor β-induced gene, TGFBI, on human chromosome 5 (5q31). TGFBI is activated by corneal trauma, leading to the overproduction of transforming growth factor β-induced protein (TGFBIp), which is the main component of corneal opacities [2].

GCD2 is characterized by hyaline and amyloid deposits in the basal epithelial and stromal layers of the cornea. Patients are often asymptomatic but may experience glare [3]. Granular opacities typically appear earlier than lattice opacities and are preferentially found in the superficial layers of the stroma, while lattice deposits are deeper [3]. As described by Kobayashi A et al., lesions appear on the slit-lamp examination as multiple, round, sharply demarcated gray-white deposits separated by clear corneal areas [4]. In vivo confocal microscopy (IVCM) images show focal deposition of highly reflective granular materials in the basal epithelial layer and highly reflective granular materials with irregular edges in the superficial and middle stroma [4]. Thinned subbasal nerve fibers are found between these deposits [5], and the endothelium remains unaffected [6].

## Case presentation

A 35-year-old Greek woman, recently diagnosed with epilepsy and with no other medical conditions, presented with complaints of blurry vision while wearing her contact lenses, especially at night. The best-corrected visual acuity (BCVA) was 20/20 in both eyes (Snellen Chart).

**Figure 1:**
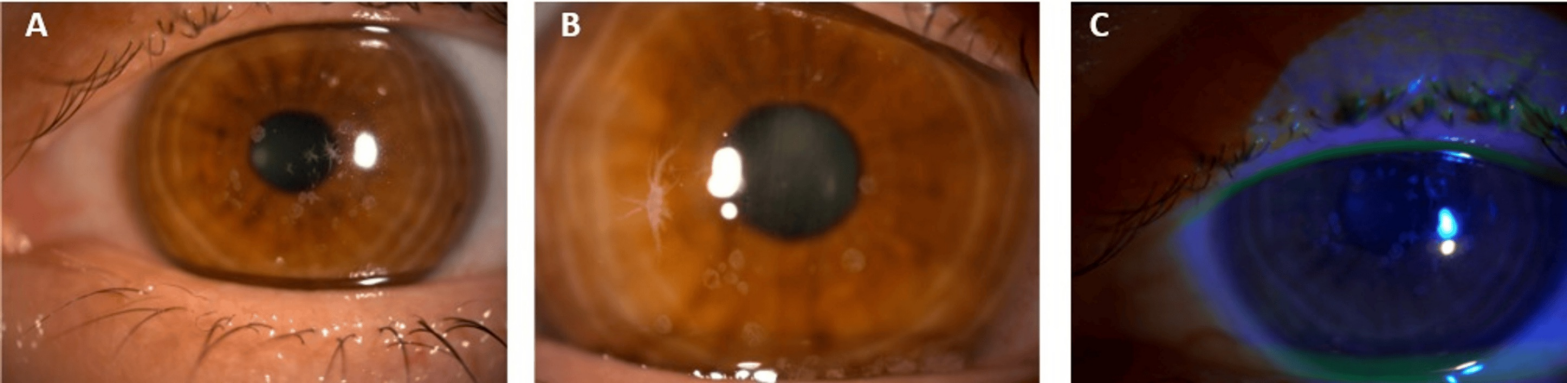
Slit lamp photographs of the cornea showing round or stellate gray-white opacities in the central cornea. (A) Right eye: stromal opacifications in the central cornea. (B) Left eye: stellate opacifications in the central cornea. (C) Right eye examined in blue light after local instillation of fluorescein.

During the slit-lamp examination, multiple white-gray round and stellate opacifications were observed in the central cornea of both eyes without fluorescein staining (Figure 1).

**Figure 2:**
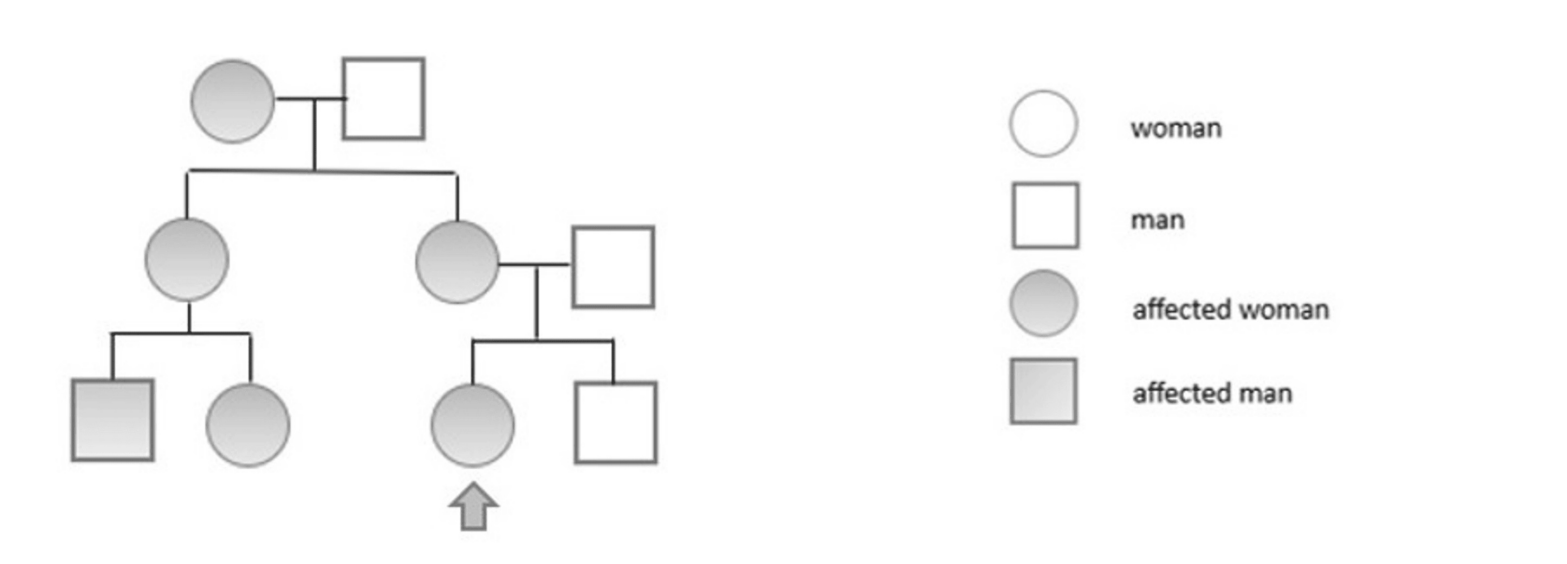
Pedigree of the patient. The arrow corresponds to the patient.

Upon discussion with the patient, similar ophthalmic conditions were observed in her mother and grandmother, leading to the establishment of a pedigree (Figure 2).

**Figure 3:**
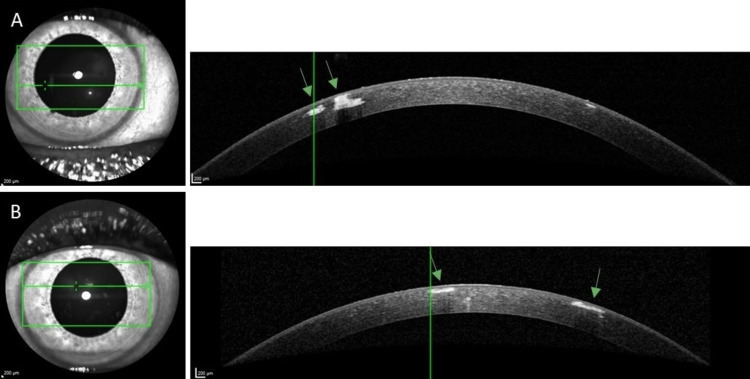
Corneal OCT (Heidelberg Engineering OCT Spectralis). Arrows show hyperreflective opacities in the anterior stroma. (A) Right eye (B) Left eye. OCT: Optical coherence tomography.

Corneal optical tomography (OCT, Heidelberg Engineering OCT Spectralis) showed hyperreflective defined areas in the anterior stroma corresponding to the opacified areas (Figure 3).

**Figure 4:**
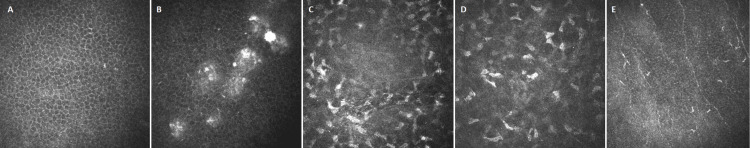
In vivo confocal microscopy (IVCM) images of the different layers of the cornea (Heidelberg Engineering HRT3). (A) Left eye (depth 0 μm): Superficial epithelium, as well as endothelium (not shown here), were normal in both eyes. (B) Right eye (depth 5 μm): In the basal epithelial layer, focal deposition of highly reflective granular material was observed. (C) Right eye (depth 22 μm): Irregular-shaped clusters of hyperreflective granular material were observed at the level of the superficial stroma. (D) Left eye (depth 61 μm): Irregular-shaped clusters of hyperreflective granular material were observed at the level of the superficial stroma. (E) Left eye (depth 24 μm): The corneal nerves appeared thinned and slender.

Various layers of the cornea were studied using the Heidelberg Engineering Heidelberg Retinal Tomograph 3 (HRT3). Clusters of hyperreflective granular material were observed at the level of the superficial stroma and in the basal epithelial layer (Figure 4).

This observation, combined with the family history, suggests granular corneal dystrophy type II, also known as Avellino corneal dystrophy.

Following the clinical examination, artificial tears were prescribed to the patient to hydrate the cornea and reduce the risk of erosion. As the patient's vision was spared, no other treatment was required. Regular follow-up was advised to the patient who returned to her home country.

## Discussion

Most stromal dystrophies are difficult to distinguish clinically and require histopathological or genetic testing for an accurate diagnosis. The clinical significance of distinguishing between different types of corneal dystrophies is crucial as an accurate diagnosis helps guide appropriate treatment strategies and informs prognosis. In this case, IVCM could be a useful non-invasive alternative to the invasive tests mentioned above since the features studied by IVCM are typical of each entity.

The IVCM images of this patient are indeed consistent with Kobayashi A et al.’s description of Avellino corneal dystrophy and differ from the IVCM features of other corneal dystrophies. For example, in lattice corneal dystrophy type I, the lesions appear as reticular, highly reflective extracellular deposits in the basal epithelial layer and highly reflective branching filaments in the superficial and middle stroma [4]. On the other hand, macular corneal dystrophy is characterized by highly reflective deposits without distinct borders in the basal epithelial layer and superficial stroma. Rectilinear hyperreflective deposits are observed in the anterior stroma, and homogeneous reflective materials with dark striae of different lengths and orientations are observed in the middle and posterior stroma [4,7]. Endothelial cell characteristics differ from study to study. Micali A et al. showed bright granules and polymegathism, while the endothelium was totally normal in Kobayashi A et al.’s study, highlighting the importance of studying larger cohorts to uncover more pathological features [4,7]. In the case of Schnyder corneal dystrophy, IVCM images show hyperreflective deposits in the epithelium and needle-shaped or rectangular crystal deposits in the stroma, and damaged keratocytes characterized by the absence of nuclei visualization. The subbasal nerve plexus was either thinned or not detected at all [8,9].

The ability to visualize and quantify the extent of corneal deposits using IVCM can also aid in evaluating the effectiveness of therapeutic interventions. This is especially relevant for patients undergoing treatments such as phototherapeutic keratectomy (PTK) or deep anterior lamellar keratoplasty (DALK). PTK is usually used in corneal dystrophies for therapeutic and/or vision improvement purposes. The visual improvement after PTK is explained by the removal or density reduction of corneal opacities and the reduction of irregular astigmatism [10]. Furthermore, IVCM can be utilized to study the regeneration and repair of corneal nerve fibers post-treatment, providing insights into the recovery process and potential complications.

Another significant advantage of IVCM is its ability to detect subclinical changes in the cornea that are not visible with traditional imaging techniques. This early detection can be crucial for initiating timely interventions and preventing disease progression.

## Conclusions

IVCM has proved to be a valuable, non-invasive diagnostic tool for distinguishing various corneal dystrophies, when combined with slit lamp examination and OCT. The detailed images provided by IVCM allow for a better understanding of the microstructural changes in the cornea, which can lead to more targeted and personalized treatment plans.

Future research directions could include exploring the utility of IVCM in a larger cohort of patients with various corneal dystrophies to further validate its diagnostic accuracy and to potentially uncover new pathological features.

## References

[1] Lisch W, Weiss JS (2019). Clinical and genetic update of corneal dystrophies. Exp Eye Res.

[2] Han KE, Kim TI, Chung WS, Choi SI, Kim BY, Kim EK (2010). Clinical findings and treatments of granular corneal dystrophy type 2 (avellino corneal dystrophy): a review of the literature. Eye Contact Lens.

[3] Lin ZN, Chen J, Cui HP (2016). Characteristics of corneal dystrophies: a review from clinical, histological and genetic perspectives. Int J Ophthalmol.

[4] Kobayashi A, Fujiki K, Fujimaki T, Murakami A, Sugiyama K (2007). In vivo laser confocal microscopic findings of corneal stromal dystrophies. Arch Ophthalmol.

[5] Traversi C, Martone G, Malandrini A, Tosi GM, Caporossi A (2006). In vivo confocal microscopy in recurrent granular dystrophy in corneal graft after penetrating keratoplasty. Clin Exp Ophthalmol.

[6] Chang MS, Jun I, Kim EK (2023). Mini-review: clinical features and management of granular corneal dystrophy type 2. Korean J Ophthalmol.

[7] Micali A, Pisani A, Puzzolo D (2014). Macular corneal dystrophy: in vivo confocal and structural data. Ophthalmology.

[8] Ghazal W, Georgeon C, Grieve K, Bouheraoua N, Borderie V (2020). Multimodal imaging features of schnyder corneal dystrophy. J Ophthalmol.

[9] Kobayashi A, Fujiki K, Murakami A, Sugiyama K (2009). In vivo laser confocal microscopy findings and mutational analysis for Schnyder's crystalline corneal dystrophy. Ophthalmology.

[10] Rathi VM, Vyas SP, Sangwan VS (2012). Phototherapeutic keratectomy. Indian J Ophthalmol.

